# Prospectus for 1896

**Published:** 1895-12

**Authors:** 


### EDITORIAL.

### PROSPECTUS FOR 1896.

WITH this number we close the sixteenth volume of the JOURNAL OF COMPARATIVE MEDICINE AND VETERINARY ARCHIVES, and in so doing it is with a conscientious feeling that we have discharged with fidelity the many exacting duties that fall to the editorship of a scientific journal. We shall have but little to say as to how well we have borne the responsibilities of such a position, but express our warmest gratitude for the many kind expressions of approval of our efforts that we have received from many of our readers and for the valuable assistance they have rendered us by their contributions, reports, and current information during the year.

The JOURNAL for the new year of 1896 will be continued under the same general direction, though there will be changes on the editorial staff and ownership. As in the past, it will be fearless, independent, the organ of no association, school, or organization, and its editorials will be outspoken and direct upon every question that pertains to the domain of the veterinarian. Every movement that has for its sole aim the aggrandizement of the profession will receive our most sincere and unqualified support, all others our criticism and condemnation. Every college movement that has for its aim the higher equipment of her students will be supported and encouraged at all times and our influence loaned to aid it in every way. Every attempt to lower the standard of veterinary education will be watched, criticised, and defeated if possible. Association work, local, State, and national, will be carefully watched and encouraged in every way within our power. Control-work at home and abroad will be kept pace with, and every avenue of information will be zealously watched that our readers may be kept fully informed of every advance along the line. The best productions from the best writers will be drawn from to aid our readers in the better performance of their work, and abstracts from foreign journals, reports of cases, correspondence, etc., be freely drawn upon to add to the interest of our pages. In every direction the JOURNAL will strive to be foremost in its reading-matter, most complete in news and general information, and a power for good on behalf of the profession in every channel opening before it. It will spare none who attempt to misuse their power and position, but with all our force searchingly criticise the wrongdoer in every sphere and place. Our aim shall constantly be to lead veterinary journalism in America.

